# P–N Nanoporous Silicon Fabrication Using Photoelectrochemical Etching and Ultrasonic Vibration and Liquid-Phase Bonding for Optoelectronic Applications

**DOI:** 10.3390/mi17010073

**Published:** 2026-01-04

**Authors:** Chao-Ching Chiang, Philip Nathaniel Immanuel

**Affiliations:** 1 Department of Mechanical Engineering, Asia Eastern University of Science and Technology, New Taipei City 220303, Taiwan; 2 Department of Chemical Engineering, Ariel University, Ariel 407000, Israel; nathaniel.philip@gmail.com

**Keywords:** nanoporous silicon diode, photoelectrochemical etching and ultrasonic vibration, liquid-phase bonding, quantum efficiency, thermal annealing

## Abstract

We systematically investigated the optical properties of P-N nanoporous silicon (NPS) diodes fabricated using photoelectrochemical etching and ultrasonic vibration (PEEU), followed by liquid-phase bonding and thermal treatment. Ultrasonic vibration during etching promoted uniform pore formation by enhancing reactant diffusion and suppressing hydrogen bubble accumulation, while laser-induced photocarriers improved etching selectivity, facilitating the formation of NPS with pronounced quantum confinement. The fabricated NPS devices exhibited significantly enhanced photoluminescence (PL) and electroluminescence (EL) properties, with an average external quantum efficiency of 7.3% at a bias of 10 V. Subsequent liquid-phase bonding and thermal annealing further enhanced structural stability and interface quality, resulting in an 180% increase in PL intensity. These results demonstrate that the combination of PEEU with liquid-phase bonding and thermal annealing yields a versatile approach to tailor the optical and electrical properties of P–N porous silicon nanostructures for high-performance light-emitting diodes and quantum-confined silicon photonics, highlighting the critical role of process-induced nanostructures and thermal modifications in device performance.

## 1. Introduction

The earliest documented observation of porous silicon (PS) was made by Uhlir in 1956 [1] while conducting electrochemical silicon polishing experiments at Bell Labs. He observed the formation of a black porous film on a silicon wafer immersed in a hydrofluoric acid (HF) solution, now known as the PS layer. This film features a nanoscale controllable pore structure and has distinct optical properties compared to other silicon-based materials. By controlling the electrochemical etching parameters, the pore size can reach the nanoscale, confining the movement of electrons and holes and triggering quantum confinement effects [2], which widen the energy gap and enable visible light emission. These characteristics have made PS an important area for nanophotoelectric research, showing promise for applications in optoelectronic devices [3], sensors [4], energy storage [5], biomedicine [6], and micro-electromechanical systems [7].

Currently, PS is primarily fabricated using electrochemical etching. An external voltage is applied to a silicon substrate submerged in an HF-based etchant, causing silicon atoms to react with fluoride ions to form volatile SiF_4_ or soluble H_2_SiF_6_, gradually developing a porous structure. The pore morphology, distribution, and size can be controlled by manipulating the current density, HF concentration, and solution additives (such as ethanol or deionized (DI) water). This approach offers the benefits of rapid processing and simpler equipment, allowing for fine control over the porosity, thickness, and nanoscale structure of the PS to suit application requirements. However, in conventional electrochemical etching, the resulting porous silicon layer (PSL) structure is heavily dependent on the process variables, making uniform etching and consistent pore morphology difficult [8].

In the 1990s, Gösele (Duke University) and Canham (University of Birmingham, UK) proposed a new bandgap theory model [9,10], pointing out that PS luminescence stems from quantum confinement within its nanoscale silicon crystal walls. This effect transforms indirect-bandgap silicon into a “direct-bandgap-like” material, enabling efficient electron–hole recombination and luminescence. Their theory was confirmed by the observation of strong visible light photoluminescence (PL) in PS at room temperature. Silicon bonding methods for silicon-to-silicon substrates are generally categorized as direct or plasma bonding: in direct bonding, silicon is immersed in an HF solution for tens of seconds to remove the surface oxide layer, and then irradiated with ultraviolet (UV) light (B-100AP, Analytik Jena US LLC, Upland, CA, USA) in air before low-temperature heat treatment. Although the bonding process is simple, achieving large-scale effective bonding remains challenging. Plasma bonding uses plasma treatment to bond silicon layers together [11]. The ability to fabricate components with improved performance depends on accurately managing the plasma treatment variables, including the processing time, the input power, and the working pressure. In addition, high plasma energy can damage surfaces, seriously affecting optoelectronic device performance and bonding integrity [12].

For silicon-based electroluminescent (EL) devices, carefully designed P–N NPS structures allow for carrier injection and recombination luminescence [13]. In energy applications, NPS is widely used in supercapacitors due to its high specific surface area and adjustable pore size [14]. PS devices are also common in optoelectronics [15,16]. Furthermore, combining P-type and N-type PSLs to form a P–N heterojunction enables electron and hole injection, generating recombination under an external bias, resulting in highly visible EL. However, it is difficult in conventional high-temperature bonding or plasma bonding techniques to control interface defects and oxide layer thickness, reducing carrier recombination efficiency and luminescence stability [17].

Negative differential resistance (NDR) [18,19] is a nonlinear current–voltage (I–V) characteristic where, within a specific voltage range, an increase in voltage leads to a decrease in current (dI/dV < 0). This phenomenon violates the linear conduction behavior of Ohm’s law, but is common in nanoscale materials and devices, signaling non-equilibrium carrier transport mechanisms such as quantum tunneling, Coulomb blocking, or band alignment modulation [20]. Accurate, repeatable control of electrochemical etching parameters for silicon is critical. The NDR’s unique behavior is useful for applications in memory and oscillators [21]. The NDR found in the Esaki diode comes from the tunneling current of the band overlap in highly doped P–N junctions.

In a previous study, Chiang et al. investigated trends and variations in the NDR phenomenon in P–N PS diodes fabricated using PEEU [13,22,23]. The electrochemical etching process reshapes the silicon crystal into a matrix containing nanocrystals and nanoscale pores, which enhances quantum confinement. Such a porous configuration allows the PS layer to operate as a nano capacitive zone.

PEEU can be tuned for different applications. Ultrasonic vibration helps clear bubbles from the photoelectrochemical etching area, enhancing the bandgap energy absorption (BEA) reaction [22] and the overall PL performance of the nanocrystalline porous silicon (NC–PS) layer. In NPS devices, the NDR effect observed in the single-layer PS is affected by the structural state and etching parameters. Using PEEU results in highly uniform nanoscale pores, and long-wavelength lasers enhance photogenerated carrier generation and etching efficiency. The resulting device displays clear EL under bias drive, and stable NDR between 0 and 5 V, confirming quantum confinement in the porous structure. The structure of the diode contains three main parts: a metal film used as the surface electrode, an NPS layer beneath it, and a back contact that operates as a planar cold cathode [23]. The quantum size of the NPS structure enhances tunneling, speeding up electron transport through the layer and boosting luminescence. 3-Aminopropyltriethoxysilane (H_2_N–(CH_2_)_3_–Si(OC_2_H_5_)_3_, APTES) (99%, Wego Chemical Group, Great Neck, NY, USA) is a commonly used coupling agent for connecting chemically reactive amino groups on Si and Si substrates [24,25,26], but conventional organosilane bonding requires pre–oxidation [27,28] (thermal oxidation) to produce a silicon dioxide layer at the bonding interface, which can hinder luminescence performance of the P-N NPS device. As reported in earlier work [29], conventional electrochemical etching methods are capable of generating red photoluminescence from NC–PS. While this enhances the electroluminescence intensity to some extent, the overall efficiency remains insufficient for practical application [30]. Since pore size distribution affects luminescence, this work continues to use PEEU for controlled PS pore sizing and uniform NPS areas to improve performance

This study details the fabrication of P–N PS diode devices by combining PEEU with liquid-phase bonding techniques. By precisely controlling the pore size and creating chemical bonds (Si–O–Si) with the surface, this method enhances interfacial carrier injection and quantum confinement luminescence efficiency. This approach holds promise for the manufacture wavelength-tunable, integrated silicon-based light-emitting devices (LEDs) at low temperatures. Initially, PEEU forms NPS nanostructures, thereby broadening the visible-light PL wavelength range while minimizing electron confinement due to quantum size effects. Liquid-phase bonding, performed in anhydrous ethanol (EtOH) and APTES prevents the formation of a thick silicon dioxide layer on the PS surface, preserving device performance [24,31].

For high-performance electroluminescent devices, the P–type and N–type PS layers are bonded with anhydrous EtOH and APTES [32,33]. Bonding conditions and duration are precisely controlled to ensure strong adhesion. Once bonded, the PS diode is assembled, with PSL thickness maintained at the nanometer scale. The NC–Si layer is formed by photoelectrochemical etching, followed by deposition of the metal electrode layer on the PSL surface via electrochemical bonding and the application of a glass film to protect the device from environmental contamination. X-ray photoelectron spectroscopy (XPS), PL spectroscopy, field-emission scanning electron microscopy (FE–SEM), and transmission electron microscopy (TEM) are employed to characterize the material and observe the interface and crystal diffraction of the bonded NPS. These techniques help enhance etching and bonding parameters for better process control and stability, advancing NPS diode device fabrication. Ongoing optimization of etching control, surface modification, and bonding processes will contribute to the development of more efficient silicon-based LEDs and provide a viable pathway for future photodiode technologies.

## 2. Experimental Procedure

The photoelectrochemical etching system used in this study consists of three components: an ultrasonic oscillator (Model: LEO–803H, maximum capacity: 2L, frequency: 120 kHz, LEO ULTRASONIC Co., Ltd., New Taipei City, Taiwan); a power supply system (model ABM-PR8363) (ABM, New Taipei City, Taiwan) to provide the current and voltage required for the experiments; and a laser irradiation platform for photoexcitation of silicon samples.

The silicon–based materials used as test samples included boron–doped (B) p–type and arsenic–doped (As) single–crystal silicon wafers (100) from Innotech Corporation, Miaoli County, Taiwan, with resistivity ranging from 1 to 10 Ω·cm, B concentration of (1.34–4.6) × 10^15^ cm^−3^, and As concentration of (1–5) × 10^18^ cm^−3^ (Ingentec Corporation, Miaoli County, Taiwan), and a sample size of 5 × 5 cm^2^. The laser platform employs a 1310 nm wavelength laser with a maximum power of 20 mW. For precise laser energy control, a Glan laser polarizer with a wavelength range of 350–2300 nm (Unice 2–GL–3522-3, Edmund Optics in Barrington, NJ, USA) was installed along the optical path, with a PM100D laser power meter (Thorlabs, Newton, NJ, USA) integrated for real-time power measurement.

**Figure 1:**
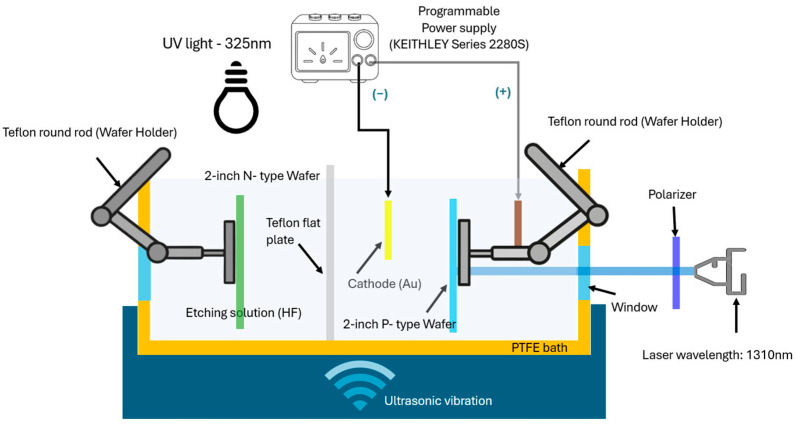
A depiction of the experimental configuration, including the photoelectrochemical etching system and the ultrasonic vibration unit.

This system, shown in Figure 1, overcomes the shortcomings of the conventional electrochemical etching processes, particularly the difficulty in controlling the size and reproducibility of PS structures. After bonding, measurements of the photoluminescence trends of the NPS components confirmed enhancement of the BEA electrothermal reaction [22], enabling precise control of the EL properties. Any contamination on the specimen, such as particulate matter, oily residues, or metal ions, can compromise the consistency of the process. This often leads to uneven etching and distorted experimental results. The single-crystal silicon surface of a 2-inch silicon wafer was first cleaned in a Piranha solution using the sulfuric peroxide mixture (SPM) cleaning process. This was followed by RCA-1 cleaning (NH_4_OH:H_2_O_2_:H_2_O = 1:1:5 by volume, for 180 s) and RCA-2 cleaning (HCl:H_2_O_2_:H_2_O = 1:1:6 by volume, for 300 s) using a boiling solution. Finally, the samples were immersed in diluted HF (DHF; 1:100 by volume, for 10 s) to completely remove native surface oxides and residues. The samples were then dried in a desiccator and stored in a filter vacuum desiccator (model: 550, Kartell^®^, Noviglio, Italy) prior to PEEU.

The PEEU process was performed in a Polytetrafluoroethylene (PTFE, commercially known as Teflon) bath with an electrolyte volume of 500 mL. The etching solution consisted of a mixture of 49.5% HF and 99.5% EtOH (volume ratio 1:2). The voltage was 10 V, the current density was 100 mA/cm^2^, and the etching time was 15 min. Etching was performed using a two-electrode electrochemical method, with a gold electrode (Au purity 99%, conductivity: 63.01 × 10^6^ S·m^−1^, 20 °C) serving as the cathode. The etched sample area was 1.75 × 1.75 cm^2^. The specimen was secured in the bath using a Teflon holder. After PEEU was completed, the specimen was liquid-phase-bonded directly in the bath to prevent direct exposure of the specimen to air, which could lead to recontamination.

The thermoelectric effect, first observed experimentally as a thermal current by Thomas Johann Seebeck in 1801 [34], causes problems in traditional electrochemical etching, leading to uneven pore size distribution, high interface roughness, and structural defects, which degrade the stability and efficiency of silicon-based optoelectronic devices. The authors previously modified the PEEU process by leveraging the selective reaction of photogenerated carriers and ultrasonic vibration, to promote efficient etchant reactions within the silicon pore structure, achieving a balanced etching effect. This method effectively controls the formation of nanoscale pores, reduces surface defect density, and improves the structural uniformity and reproducibility of the PSL, thus overcoming the difficulties of conventional electrochemical etching in nanoscale control.

Laser irradiation in an electrochemical environment encourages the formation of B–Si (boron–silicon) bonds or B–Si aggregates, leading to the creation of a local chemical shielding layer. This layer reduces the direct chemical reaction between HF molecules or fluorine ions and silicon atoms at the microscopic level, resulting in local etching rate inhomogeneity. When the long-wavelength laser fully penetrates the silicon wafer, it generates photocarriers (electrons and holes) and induces the BEA effect, redistributing boron atoms and promoting more B–Si complexes or altering electronic state densities. These localized chemical/thermal shielding and BEA effects result in the coexistence of the nucleation inhibition zones in the early etching phase and surrounding active etching regions, influencing pore size, distribution, and porous layer thickness [23]. The coupling of thermal and optical excitation not only alters the spatial distribution of dopant atoms but also changes the surface chemical state, causing localized temperature increases and promoting surface oxidation. These changes interact to shift the current density distribution, preferentially concentrating holes at field-enhanced or defect locations, thereby promoting the growth of deep, narrow channels in those locations (tip field effect). The NPS morphology thus reflects a dynamic equilibrium driven by light, heat, and chemical reactions. Etching conditions can be controlled by varying the laser power supplied to the silicon surface.

Ultrasonic vibration plays two crucial roles in this system. First, it removes hydrogen bubbles and reaction byproducts generated in the etching zone through cavitation and microjet effects, thereby preventing uneven etching caused by bubble shielding. Second, it boosts microscale fluid convection, improving mass transfer, increasing the transport efficiency of HF and intermediate products. This improves electrochemical stability and uniform pore formation during PEEU. The pulsating pressure waves induced by ultrasound promote bubble detachment and redistribution within the BEA (photoinduced electron–hole depletion) region [23], resulting in more continuous and homogeneous NC–PS structures in the experiments [13]. At this stage, the PS film becomes saturated in the HF solution. The chemical reactions governing PS film formation are summarized in Equations (1)–(3) [1]. Under laser irradiation, boron–silicon (B–Si) bonds accumulate near the silicon surface and in deeper layers, trapping some B atoms locally, lowering the etching rate [29]. Meanwhile, oxidation during photoelectrochemical etching generates hydrogen gas, as shown in Equation (4) [35]. (2 − n) is not a standard chemical substance; rather, it represents the amount of HF, which varies depending on the value of n, corresponding to the number of electrons generated during the reaction. Specifically, n denotes the number of electrons produced, while 2 − n indicates the number of remaining HF molecules. In this way, the reaction should be understood as a half-reaction describing the process of electron generation when silicon reacts with HF, rather than as a fixed chemical equation [1].

The overall microscopic formation mechanism can be summarized as follows: holes remain the dominant carriers in NPS growth, and their spatial and temporal distribution is determined by the photoexcitation rate, doping profile (boron), local temperature, and mass transfer conditions. Localized B–Si aggregates and photothermal activation temporarily inhibit etching in certain areas, but ultrasonic vibration, by enhancing bubble removal and mass transfer, mitigates these inhibitory effects, ensuring pore structure uniformity. In the equation, s denotes a solid, l  denotes a liquid, g denotes a gas, and aq denotes an aqueous solution.


(1) $$Si(s)+2HF(aq)+(2-n)\to SiF_{2}(aq)+2H^{+}(aq)+ne^{-};$$

(2) $$SiF_{2}(aq)+2HF(aq)\to H_{2}SiF_{6}(aq);$$

(3) $$SiF_{4}(aq)+2HF(aq)\to SiF_{6}^{2-}(aq)+2H^{+}\left(g\right);$$

(4) $$Si(s)+6HF(aq)\to H_{2}{SiF}_{6}(aq)+4H_{2}(g)↑.$$

The PEEU mechanism drives holes across the depletion layer into the silicon surface, thereby activating the BEA process [23]. By amplifying the photon–boron interaction, PEEU contributes to a stronger BEA effect. The combination of photoelectrochemical etching and ultrasonic vibration promotes the formation of a more uniform and stronger emission region. However, excessive laser power can inhibit the etching of the P–type PSL and induce B excited–state absorption, preventing B atoms surrounded by silicon atoms from transitioning to higher energy states. Furthermore, mutual attraction between B atoms and internal silicon electrons (B–e^−^ ↔ Si–e^−^) alters the external Si–Si bonds [30], an effect compounded by the formation of volatile SiF_2_ species, which further modify these bonds. This process simultaneously releases electrons and hydrogen bubbles, thereby enhancing the overall photoelectrochemical reaction.

**Figure 2:**
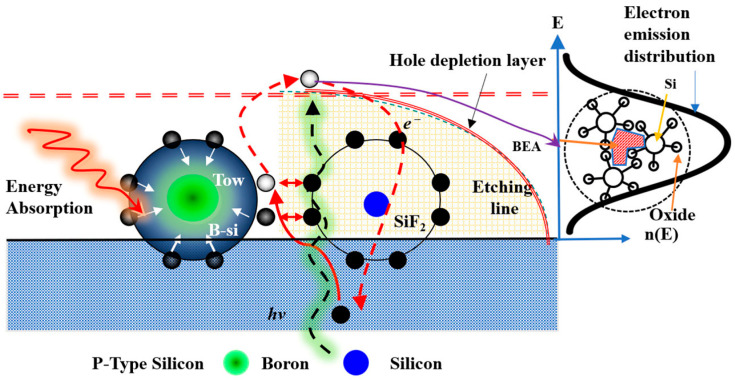
Proposed mechanism of laser-assisted photoelectrochemical etching of p-type silicon showing carrier dynamics and BEA effect [23].

As illustrated in Figure 2 [23], the reaction mechanism involves incident long-wavelength laser light penetrating the silicon layer and exciting B atoms, which leads to localized energy absorption and the formation of B–Si bonds. The absorbed energy disperses outward within the energy diffusion region, promoting charge redistribution across the depletion layer and enhancing hole–electron separation, a process known as the BEA effect. In this region, photogenerated holes migrate toward the Si/HF interface, where a redox reaction occurs, releasing H_2_ and forming SiF_6_ species. The temporary accumulation of B–Si clusters shields nearby Si atoms, suppressing localized etching. Outside this affected area, HF-driven dissolution predominates. Ultrasonic vibration assists by removing bubbles and reaction by products, contributing towards a consistent and stable etching environment.

Additionally, laser-induced photogenerated holes migrate toward the surface within the depletion layer, triggering the BEA reaction, which supports carrier separation and accelerates the redox reaction at the Si/HF interface. The formation of H_2_ and dynamic surface oxide layers introduce local surface energy variations, resulting in etched pits. The application of ultrasonic vibration helps eliminate bubbles, enhances the transport of reactants and products, and stabilizes both the current distribution and the etched morphology, as described in Equation (3). Collectively, the reaction mechanism encompasses five stages: (1) laser penetration and B atom energy absorption (energy absorption), (2) B–Si bond formation and local energy diffusion (energy diffusion of B–Si clusters), (3) hole migration and BEA reaction region formation (hole migration/BEA region), (4) HF–driven redox etching (Si dissolution to H_2_SiF_6_ + H_2_), and (5) ultrasonic vibration-assisted bubble detachment and structural homogenization (acoustic-assisted stabilization). This multi–faceted interaction among light, electricity, chemistry, and ultrasound establishes a dynamically balanced environment for NPS growth, resulting in a more uniform pore size distribution in the NPS layer and significantly improving the density and stability of the light-emitting active region.

**Figure 3:**
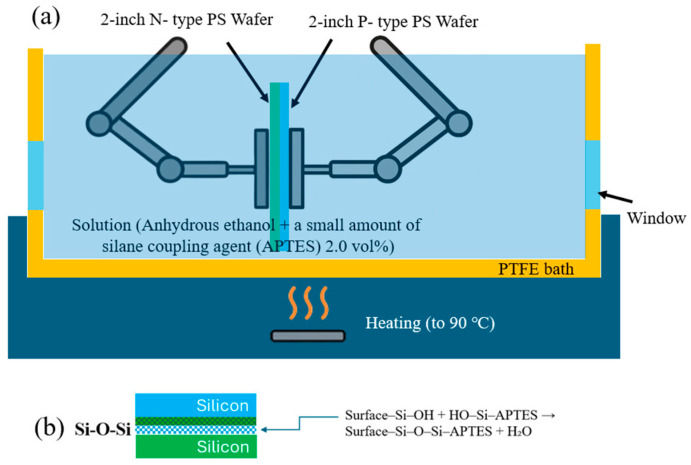
(**a**) An illustration of the liquid-phase bonding of PS wafers in APTES after the photoelectrochemical etching step; (**b**) the chemical structure representing P–N liquid-phase bonding.

Bonding was conducted in the etching tank using anhydrous EtOH (99.5%, Nihon Shiyaku Co., Ltd., Tokyo, Japan) and APTES, with the latter diluted in EtOH at a volume ratio of 100:2, as shown in Figure 3a. This methodology represents the most effective approach for the formation of chemical bonds within the tank without exposing the sample to external environment contaminants.

Step 1: After HF etching is completed, the etching solution is drained, and DI water is rapidly introduced to fill the tank. This rinsing cycle is repeated three times (each cycle lasting 30 s) to remove residual HF etching solution from the PS surface. Step 2: A substantial volume of DI water is added for a brief 30 s soak, followed by prompt drainage to remove residual HF. During drainage, anhydrous EtOH is immediately injected (at least three times) to displace the DI water. Step 3: Preparation of a silane solution (APTES concentration: 2.0 vol%) in anhydrous EtOH. Step 4: Immerse the PS specimens in this APTES-containing anhydrous EtOH bath for 10 min to facilitate adsorption of the silane molecules onto the porous surfaces. Align the two PS specimens (still immersed in the solvent bath) using Teflon rods, so that the etched surfaces are facing each other. Apply uniform pressure of 20 kPa using a linkage mechanism to prevent cracking. Step 5: Maintaining submersion, heat the specimens in a furnace at 90 °C for 5, 10, and 15 min, respectively, for three sets. Heating promotes APTES condensation (Si–O–Si), removes residual DI water and anhydrous EtOH, and enhances silane condensation and interfacial bonding, as shown in Figure 3b.

APTES effectively enables chemical bonding between silicon surfaces, following a three-stage reaction process. Initially, under the influence of anhydrous EtOH solution or trace environmental moisture, the ethoxy groups of APTES undergo hydrolysis, yielding highly reactive trihydroxysilane (Si(OH)_3_), as detailed in Equation (5) [36]. This step transforms APTES from hydrophobic to polar and reactive, facilitating condensation reactions with silanol groups (≡Si–OH) on the silicon surface. Subsequently, in the presence of abundant silanol groups (typically generated by HF etching or oxidation), the Si–OH groups of APTES undergo dehydration–condensation reactions with surface Si–OH entities, to form stable covalent Si–O–Si linkages, as detailed in Equation (6) [36]. This step firmly anchors the APTES molecules to the silicon surface, creating exposed active amine groups (–NH_2_) suitable for subsequent bonding reactions. Finally, upon contact between the PS wafer and the APTES-modified silicon surface, further silicon–oxygen crosslinking (Si–O–Si) occurs, forming a chemically stable and mechanically robust bridging layer. This layer enhances the bond strength and thermal resistance between the silicon wafers. The APTES bonding mechanism is thus consolidated into three key steps: hydrolysis, condensation, and crosslinking, achieving chemical connection from the molecular to the interfacial level. This approach effectively averts silane self-aggregation due to air exposure and ensures uniform and stable interfacial bonding.


(5) $$\mathrm{Si}{\left({\mathrm{OC}}_{2}H_{5}\right)}_{3}\;(l)+3H_{2}O\;(l)\;\to \;\mathrm{Si}{\left(\mathrm{OH}\right)}_{3}\;(s)+3C_{2}H_{5}\mathrm{OH}\;(l)$$

(6) $$\mathrm{Surface}–\mathrm{Si}–\mathrm{OH}(s)+\mathrm{HO}–\mathrm{Si}–\mathrm{APTES}\left(s+aq\right)\;\to \mathrm{Surface}–\mathrm{Si}–O–\mathrm{Si}–\mathrm{APTES}(s+aq)+H_{2}O(l)$$

## 3. Results and Discussion

**Figure 4:**
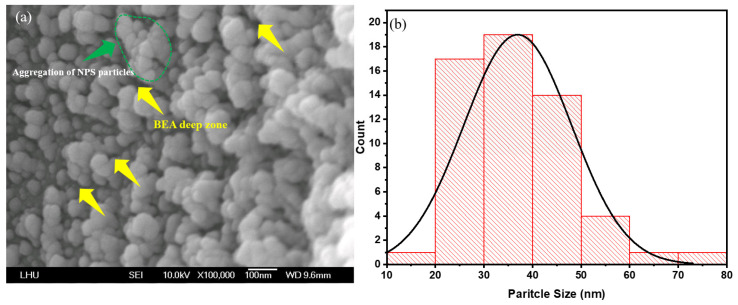
(**a**) SEM image of porous silicon fabricated by PEEU with an etching time of 5 min; (**b**) NPS pore size distribution.

**Figure 5:**
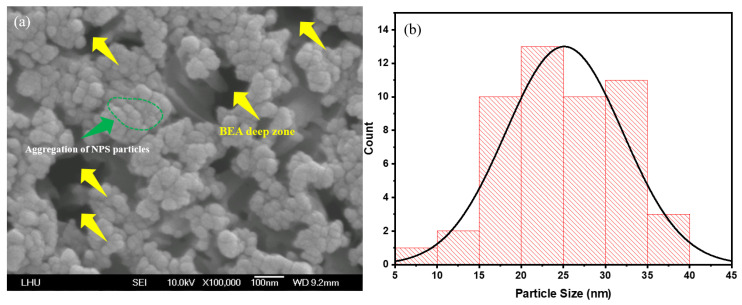
(**a**) SEM image of porous silicon fabricated by PEEU with an etching time of 10 min; (**b**) NPS pore size distribution.

**Figure 6:**
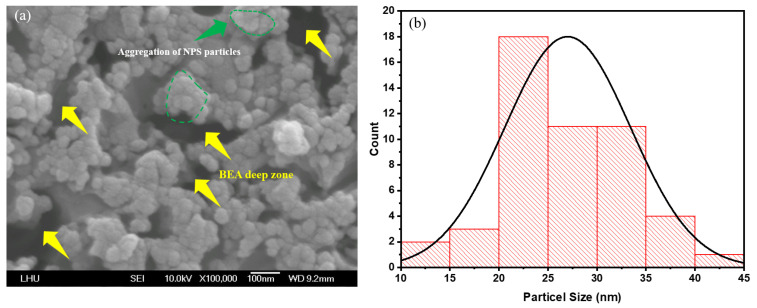
(**a**) SEM image of porous silicon fabricated by PEEU with an etching time of 15 min; (**b**) NPS pore size distribution.

First, the PSL in the laser-irradiated area was characterized to investigate the structural impact of varying laser power in this region. FE-SEM images were acquired using a field-emission scanning electron microscope (Model: JSM-6500, JEOL Ltd., Akishima, Japan) operating at 10 kV. Observations indicate that the NPS prepared by PEEU exhibits more cavity structures (Figure 4a, yellow arrow area). Upon photon energy absorption, boron atoms aggregate with silicon atoms; the silicon atoms are corroded by HF and removed from the surface. The presence of etched voids in silicon confirm that ultrasonic over-vibration effectively expels bubbles. At this juncture, the BEA reaction is initiated in the laser–irradiated region. The agglomeration of silicon within the deeper NP5 layer during photoelectrochemical etching, is mitigated by the influence of laser energy, attributable to the free carrier absorption effect. This phenomenon is evidenced by the FE–SEM images shown in Figure 4a, Figure 5a and Figure 6a, where substantial agglomerations of PS particles almost cover the entire image field. This result verifies the occurrence of free carrier absorption and provides additional evidence for the presence of borosilicate clusters (green region).

In the BEA region, large holes in the deep recessed areas suggest that they are formed on the silicon surface during the etching process. This reduces the influence of free carrier absorption and the quantum confinement effect. This can be interpreted as a consequence of variations in silicon energy bandgap under light absorption, which directly affects device current density (J_d_) in the subsequent NPS diode I–V tests. Moreover, as NPS pore size and porosity decrease, energy barriers arise for electron injections in the emission current density (J_e_) region. Enhanced quantum confinement further elevates the corresponding offset values, resulting in reduced peak and minimum I–V curve values, thereby restricting current transport and consequently reducing device luminescence efficiency. The photoluminescence behavior is strongly influenced by the distribution of pore sizes. In regions with smaller pores, the energy level separation between electrons and holes is larger, resulting in a blue shift and amplification of PL emission intensity [37].

Figure 4a, Figure 5a and Figure 6a illustrate significant changes in surface morphology and particle size distribution of the PS surface as the etching time increases from 5, 10, and 15 min. At 5 min, the surface exhibits an uneven particle distribution, with prominent nanoparticle aggregations (NPS particles) visible in localized areas, and rough pore interfaces (Figure 4a). These characteristics result from the initial stabilization phase of the etching reaction, marked by an uneven distribution of photogenerated carriers within the porous layer and the steep local HF concentration gradients, which collectively lead to pronounced etching rate variations, producing regions with larger particles and irregular morphology. The yellow arrows denote localized accumulations of large, irregular particles and deep depressions (i.e., incompletely developed etched pores). The surface is relatively rough, with sharp edges but poor adhesion between particles, as shown in Figure 4a. In contrast, the green-circled regions highlight localized particle clustering, where particles merge or are tightly packed together, forming aggregates significantly larger than average, suggests localized over-etching or redeposition.

Post PEEU etching, most of the pore sizes in the NPS structure fall within the 15–35 nm range. These nanoscale pores provide insights into the surface properties of the material and its interaction with other substances. For instance, the PL effect correlates with the pore size distribution [27]. The emission intensity varies depending on both the pore size and the degree of particle aggregation, indicating the critical role of pore distribution and density in understanding and controlling the NPS optical properties; see Figure 4b, Figure 5b and Figure 6b. This method, which couples photoelectrochemical etching with ultrasonic agitation, helps prevent hydrogen bubble accumulation, maintains uniform pores, and enhances both the PL and EL responses of NPS.

With extended etching times of 10 and 15 min (Figure 5a and Figure 6a), the FE–SEM images reveal that the nanoparticle size becomes more stable and uniformly distributed. The surface pore continuity is significantly enhanced, and particle size analysis indicates a narrower Gaussian distribution, with an average particle size of approximately 15–35 nm. This improvement can be attributed to the cavitation effect generated by ultrasonic vibration during etching, which fosters electrolyte circulation and reactant renewal within the pores. These conditions establish a more uniform reaction zone for photogenerated carriers, stabilizing nanostructure growth. Morphological differences between 10 and 15 min are marginal. The high degree of uniformity reduces aggregation, and the size distribution remains centered around the mean, indicating the approach of steady-state etching at 10 min. The 10–min sample (Figure 5a, yellow arrows) displays smaller and more uniform particles, forming a continuous, compact porous framework with fewer deeply etched regions and smoother contours. Localized aggregation (Figure 5a, green circles) is still visible, but the particle boundaries are obvious, and the size variation between clustered and surrounding regions is reduced (Figure 5a).

Similarly, in the 15-min sample, the pore and particle distributions remain uniform, with only occasional deeply etched or interconnecting channels. Aggregation is further reduced, and any microclusters that appear are shallow and concentrated near the median size range (Figure 6a).

Atomic force microscopy (AFM) was performed using an (Model: SPM-9700HT, Shimadzu, Kyoto, Japan) instrument in tapping mode with a silicon cantilever (TESPA, Bruker; spring constant 10 N/m, resonant frequency 300 kHz, tip radius ~10 nm) using the following parameters: (1) scan rate: 256 sec/frame; (2) scan ranges: 500 nm × 500 nm, 3 μm × 3 μm, and 5 μm × 5 μm. Surface roughness measurements revealed an increase proportional the etching time. The NPS etched at a laser power of 20 mW for 5 min had a surface roughness (Rq) of 1.2–3.2 nm. After 10 min of etching, Rq increased to 7.5–15 nm, and after 15 min, it further increased to 15–32 nm, as summarized in Table 1.

**Figure 7:**
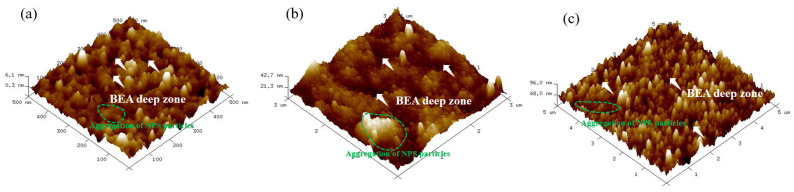
Three-dimensional AFM surface morphology of porous silicon fabricated by photoelectrochemical etching with ultrasonic assistance for different etching times: (**a**) 5 min, (**b**) 10 min, and (**c**) 15 min.

These results indicate that the absorption of laser photons by B atoms on the silicon surface facilitates the removal of outer silicon layers by HF, facilitating the formation of interconnected PS structures. In regions irradiated by 1310 nm laser light, absorption is mediated by boron rather than silicon through a free-carrier absorption mechanism. When laser photons enter silicon, boron atoms are photoexcited, creating localized energy saturation and isolation through diffusion [23]. Surrounded by silicon atoms, these excited boron atoms cannot transition to higher energy levels, so their excess energy diffuses to adjacent silicon atoms, exerting an inhibitory effect on local etching, as depicted in Figure 7.

The highest degree of PS surface uniformity was obtained at etching durations of approximately 10 to 15 min. The 5 min sample exhibited the smallest vertical scale (peak–to–valley height of about 5.8 nm) under a 0.5 × 0.5 μm scan, with an arithmetic average roughness (Ra) of 1.0–2.5 nm. In contrast, the 10–min (3 × 3 μm) and 15 min (5 × 5 μm) samples showed significantly increased roughness, with Ra values of approximately 6–12 nm and 12–25 nm, respectively, indicating that both surface height and roughness increased substantially with etching time and scan scale.

Based on the above, it can be concluded that surface roughness in the irradiated region increases with etching time. Since silicon itself does not absorb photons, only silicon–boron atomic clusters absorb the incident energy, leading to localized energy concentration and atomic agglomeration. Consequently, 1310 nm laser light which penetrates the silicon substrate primarily affects the boron atoms beneath the surface, as well as the underlying B–Si bonds, encouraging cluster formation. The contact between the silicon surface and the etching solution polarizes the Si–F bonds, which weakens the Si–Si bonds. As the Si–H bonds on the silicon surface are subsequently broken, the polarization of Si–F bonds continues, forming SiF_2_. The atoms bonded to silicon then react with HF. This process repeats itself until the Si atoms are finally removed in the form of H_2_SiF_6_. AFM images further reveal that the surface morphology gradually evolves from an initially rough structure with distinct local protrusions to a uniform and stable nanoporous surface with increasing etching time.

The surface of the 5 min etched sample exhibits pronounced variations, with multiple high peaks of approximately 6 nm and an uneven distribution, corresponding to the particle aggregations and BEA deep zones observed in the FE-SEM images (Figure 4a, Figure 5a and Figure 6a). At this early stage, the etching reaction has not yet stabilized leading to uneven distribution of photogenerated carriers and HF concentration. Localized rapid etching and redeposition result in high surface roughness. Additionally, ultrasonic cavitation may induce microbursts and localized collapses, contributing to irregular surface heights.

In the sample etched for 10 min, the surface morphology becomes distinctly flatter, with protrusions ranging from 20 to 40 nm, and a noticeable reduction in the roughness compared to the 5 min sample. FE–SEM images reveal a uniform particle distribution and continuous pores, suggesting that ultrasonic vibrations have enhanced etchant renewal and balanced the etching reaction within the pores. At this stage, etching approaches a steady state, and a continuous porous network begins to form. In the sample etched for 15 min, there is a slight increase in surface height fluctuations to approximately 50–100 nm, but the overall uniformity is maintained. FE–SEM observations confirm a stable particle size distribution. Slight regrowth or expansion of the pore walls has occurred due to continued etching, leading to a trivial increase in surface roughness. The pores remain continuous and compact, indicating that the etching kinetics are approaching equilibrium. Overall, these observations suggest that ultrasonic–assisted photoelectrochemical etching demonstrably improves fluid flow and reactant renewal within the pores, stabilizing the etching process. Optimal surface morphology and nanostructure uniformity are achieved after around 10 min of etching. FE–SEM and AFM analyses demonstrate that this combined PEEU enhances both etching uniformity and pore stability corroborating theoretical models that associate ultrasonic with steady-state etching, thereby improving the controllability and reproducibility of nanopore structures [23].

**Figure 8:**
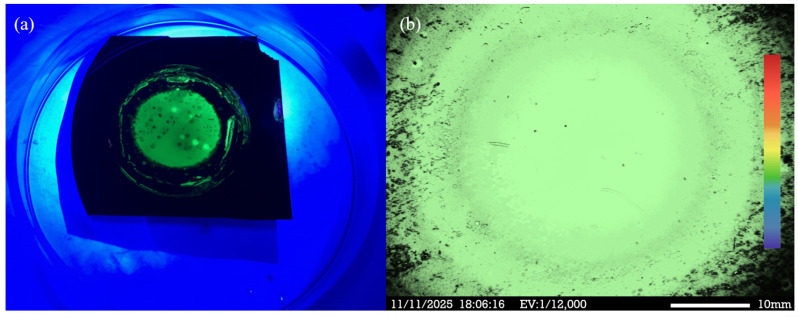
(**a**) Photograph of green emission upon excitation with 325 nm LED source; (**b**) The PS material exhibits green PL emission after undergoing PEEU.

During photoelectrochemical etching, laser interaction triggers free–carrier absorption, leading to efficient recombination of electron–hole pairs within the quantum–confined structure, and enhanced PL. The porous nanocrystal formed by the PEEU process increases porosity which contributes to quantum confinement. The PEEU process described in this study effectively controls the pore size and distribution in PS, ensuring uniform and appropriately sized pores to enhance the quantum confinement effect and improve PL efficiency. NPS nanodots excited using a 325 nm LED external light source produced a distinct green emission, as shown in Figure 8a. The PEEU process exhibited high controllability and reproducibility, significantly improving the emission characteristics of the NPS sample. The variation in emission intensity with pore size, underscores the critical importance of precise pore size control. The PL images in Figure 8b, obtained using a CRAIC 20/30 PV Microspectrometer with PL Imaging (CRAIC Technologies, San Dimas, CA, USA, EV: 12,000), reveal a uniform electron emission distribution, without noticeable distortion or noise. PL fluorescence microscopy of the BEA reaction zone also reveals a more uniform and intense green emission region. In summary, the PEEU process proposed in this study provides a novel and promising fabrication approach for NPS optoelectronic devices. This method not only improves the luminescence efficiency and uniformity of the NPS sample but also has the potential to enhance emission performance in electronic and optoelectronic applications.

**Figure 9:**
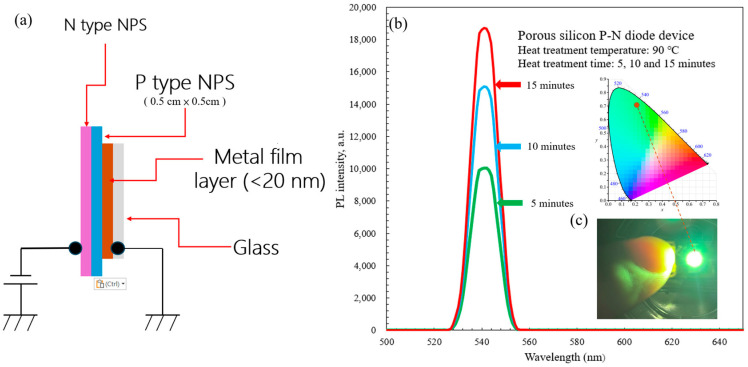
(**a**) A schematic diagram of the porous silicon P–N diode structure fabricated by PUEE with ultrasonic assistance. (**b**) PL spectra of samples heat-treated at 90 °C for 5, 10, and 15 min exhibit a green emission peak around 540–550 nm. (**c**) The inset shows the CIE chromaticity coordinates and the device’s visible green EL emission under forward bias.

The structure of the P–N NPS diode fabricated in this study is illustrated in Figure 9a. From top to bottom, the device comprises an N-type NPS layer, a P-type NPS substrate, a metal thin film (<20 nm), and a glass substrate. Ultrasonic vibration during etching promotes electrolyte renewal and bubble removal, ensuring uniform pore formation and stable pore walls. The resulting NPS layer exhibits pore diameters and silicon wall thicknesses on the order of tens of nanometers, enabling quantum confinement effects that generate visible light. A thin (less than 20 nm) gold (Au) layer serves as the ohmic contact for current injection into the P–N junction and the assembly is fixed onto a glass substrate to enhance mechanical stability and prevent environmental contamination. Under forward bias, electrons from the N-type layer recombine with holes in the P-type NPS layer within the nanocrystalline silicon walls, to emit visible-light EL dominated by quantum confinement effects.

After heat treatment (90 °C for 5, 10 and 15 min), the P–N PS diode samples fabricated via the PEEU process exhibited distinct peaks in the PL spectrum at approximately 540–550 nm, corresponding to the green region. The intensity gradually increases with longer heat treatment, reaching the highest intensity for the 15-min sample. PL spectra were measured using a fiber-optic spectrometer (Model: FluoroMax Plus, HORIBA Scientific, Irvine, CA, USA) with an integration time of 20 ms. This indicates improved structural stability and optimal recombination efficiency, as shown in Figure 9b. These results are consistent with the FE–SEM and AFM analyses. PEEU produces uniform nanoscale porosity and reduced defects, improving surface uniformity. When the etching time is around 10–15 min, the PS layer reaches steady-state growth, the pore size is approximately 15–30 nm, with pore wall thicknesses suitable for quantum confinement. Consequently, the wave functions of electrons and holes are confined in the nanoscale space, leading to an increased energy gap and a blue shift in the emission wavelength.

This phenomenon is consistent with the quantum confinement effect model proposed by Canham and Gösele [9,10], indicating that nanocrystalline silicon may attain a quasi-direct bandgap, supporting efficient radiative recombination. Furthermore, heat treatment (90 °C, 5–15 min), which removes some surface-adsorbed hydrogen and water molecules, promotes Si–O–Si bond formation, stabilizing the PS surface structure and reducing non-radiative recombination defects, further enhancing PL intensity. Shorter treatments (5 min) yielded more hydrogen terminations (Si–H) and uneven pores, favoring non-radiative recombination. Longer treatments (10–15 min) led to a higher proportion of Si–O–Si structures, lower surface trap density, and an increased probability of radiative recombination. EL imaging confirms visible light emission from the P-N NPS structure (Figure 9c, insert image). When a current is applied, electrons move from the N–type region into the porous layer to recombine with holes from the P–type region within the PS nanocrystal walls, generating green light. The CIE chromaticity diagram (Commission Internationale de l’Éclairage) shows that the emission falls in the green region (approximately 540 nm), consistent with the PL peak, indicating that the emission mechanism originates from quantum confinement effects within the NPS, as shown in Figure 9c. CIE color coordinates were determined by analyzing the PL emission spectrum shown in Figure 9b to estimate the corresponding color coordinates. By digitizing the image and mapping pixel positions along the wavelength axis (500–650 nm), the PL peak wavelength of the porous silicon P-N diode (heat-treated at 90 °C for 15 min) was determined to be 541.5 nm. Based on the CIE 1931 color matching function, the estimated chromaticity coordinates were X ≈ 0.574, Y = 1.000, and Z ≈ 0.135, with an uncertainty of approximately ±1 nm in the peak wavelength.

The combined PL and EL results demonstrate that the ultrasonic-assisted photoelectrochemical etching process effectively tunes the nanopore size and improves the optical quality of the PSL. Proper heat treatment promotes the formation of surface Si–O–Si bonds, reduces defect states, and enhances luminescence efficiency. This structure exhibits stable green light emission, highlighting its potential for application in silicon-based wavelength-tunable optoelectronic devices and integrated silicon photonic circuits.

**Figure 10:**
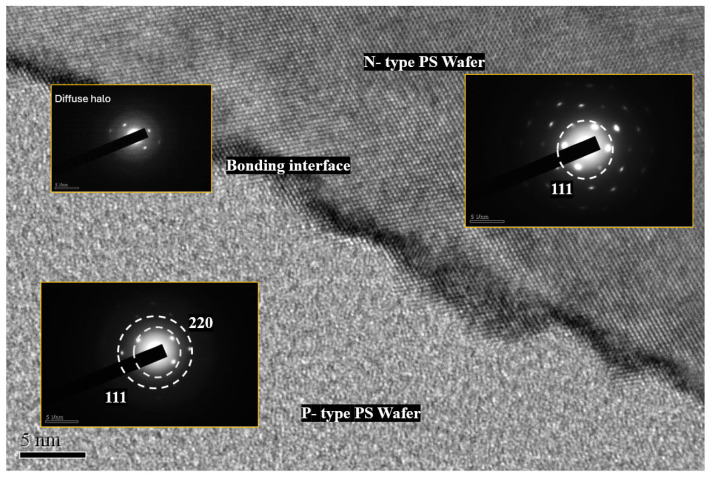
HR-TEM observations along with selected area electron diffraction results for NPS bonding.

In liquid-phase bonding, the dilute solution reacts with the silanol (Si–OH) groups on the surface of silicon dioxide to form covalent bonds. In addition, due to the activity of its functional groups, APTES can also be used to connect silicon-based materials. After electrochemical etching, the Si–OH on the silicon surface reacts to form covalent bonds. After HF or oxidation, there will be an abundance of Si–OH (silanol groups). The abundant Si–OH of APTES and the Si–OH on the surface facilitate dehydration and condensation to form covalent Si–O–Si bonds. Liquid-phase bonding integrates the P–N PSL into the PS diode element. Laser irradiation during electrochemistry reduces the etching rate to regulate PS uniformity. The PEEU and bonding steps yield strong interfaces with Si–O–Si, Si–N and C–O–Si bonds at the bonding interface in an anhydrous EtOH and APTES environment. Most internal stress can be relieved by heating the sample to 90 °C, preventing cracking in the heterojunction and ultimately achieving a defect-free bonding interface. Figure 10 presents a high-resolution TEM image (5 nm scale) of the bonding cross-section, clearly distinguishing the upper N-type PS, the central bonding interface, and the lower P–type PS, acquired using a JEOL JEM-2100F Transmission Electron Microscope (JEOL Ltd., Akishima, Japan) at 200 kV accelerating voltage with 0.2 nm point-to-point resolution.

The figure upper-right inset presents the SAED pattern of the selected area (N–type) shows discrete bright spots and short–range order corresponding to Si(111) diffraction. The lattice fringes visible in the TEM image indicate the retention of nanocrystalline Si in this region. In contrast, the SAED pattern of the central bonding interface exhibits a broad diffuse halo without distinct spots or lattice fringes, suggesting an amorphous layer composed of APTES, organic residues or structurally disordered silicon, rather than the conventional thick SiO_2_ layer. The SAED pattern for the lower left inset (P–type) displays wider, weaker rings that can be assigned to Si(111) and Si(220), with ring broadening indicating small nanocrystallites, significant orientation dispersion, or partial amorphization. The interface with the NPS is tight and free of gaps.

Overall, the broadening and decay in intensity of the SAED rings indicate that the PS sample exhibits a nanocrystalline–amorphous hybrid phase: the N-type region retains more nanocrystalline characteristics, the P-type region comprises a partially crystalline/partially amorphous mixture, and the bonding interface is primarily composed of an amorphous or organic/silane layer.

**Figure 11:**
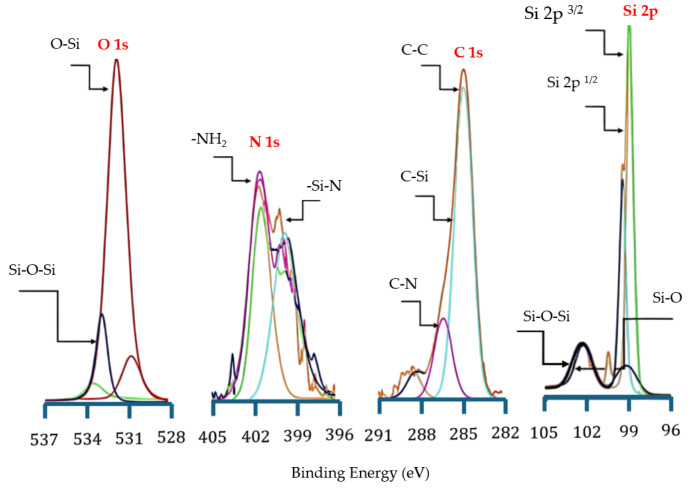
XPS spectra of surface chemistry of P–N NPS after PEEU treatment.

Further, X-ray photoelectron spectroscopy (XPS) (Model: AXIS Supra+, Kratos Analytical Ltd., Manchester, UK) was employed to analyze the surface chemical composition and bonding states of NPS samples after liquid-phase thermal treatment and verify the successful grafting of APTES molecules onto the PS surface. The observed interfacial chemical changes after the 15 min thermal treatment and pressurization are shown in Figure 11, using Al Kα radiation (1486.6 eV) at 15 kV under ultra-high vacuum, with survey and high-resolution scans of Si 2p, O 1s, and C 1s.

The N 1s spectrum exhibited a distinct nitrogen signal after bonding and thermal treatment, confirming the successful attachment of APTES molecules to the silicon surface. The main peak at 399 eV corresponds to free amine groups (–NH_2_), primarily from APTES molecules [38], while the shoulder peak at 398 eV is attributed to Si–N bonding [31]. Furthermore, the faint peak around 401 eV is likely associated with C–N or N–H cross-links that formed through intermolecular condensation of APTES. After heating, the overall N 1s intensity increased, indicating enhanced intermolecular crosslinking and improved interfacial stability.

The Si 2p spectrum displays the characteristic spin-split doublet structure: Si 2p^3/2^ (99 eV) and Si 2p^1/2^ (100.0 eV), with an energy separation of approximately 1.0 eV. The C 1s spectrum shows multiple components: a dominant peak at 284 eV corresponding to C–C bonds, a component at 283 eV corresponding to C–Si bonds, indicative of covalent bonding between the organosilane and the silicon substrate, and a peak at 286 eV assigned to C–N structures. These features further suggest the formation of a stable organosilane network within the APTES molecular layer.

Two main components were identified in the O 1s spectrum, Si–O bonds at 531 eV and Si–O–Si bonds at 533 eV, corresponding to the silicon–oxygen network formed through the hydrolysis and condensation of APTES. After heat treatment, there was a significant increase in the O 1s signal intensity, suggesting a strengthening of the Si–O–Si crosslinking structure and the removal of adsorbed water. Moreover, the three –OC_2_H_5_ groups in APTES hydrolyzed to form Si–OH groups, which subsequently reacted with –OH groups on the porous silicon surface to form stable Si–O–Si covalent bonds. The C 1s peak at 284 eV was used as the reference for energy calibration.

Altogether, the XPS results clearly demonstrate that liquid-phase bonding combined with heat treatment effectively promotes the covalent grafting of APTES molecules onto the NPS surface. The comprehensive analysis reveals that after liquid-phase thermal bonding, stable Si–O–Si and Si–N networks are formed between the APTES molecules and the NPS surface, confirming that this process significantly enhances surface grafting efficiency and chemical stability.

The I–V characteristics and emission behavior of NPS devices were systematically analyzed by measuring both the emission current and the output electron energy distribution curves. First, an NPS sample was secured within the measurement apparatus, and energy distribution (ED) measurements were performed in a vacuum chamber using an Energy Analyzer System (EAS) (EA125, Omicron Nanotechnology Inc., Taunusstein, Germany). This analysis revealed the I–V characteristics of the NPS diode, its electron emission behavior, and the corresponding NDR features, allowing for further investigation of its carrier transport and energy emission mechanisms, with measurements conducted under high vacuum (~10^−7^ mbar), anode–cathode gap of ~2 mm, electron energy range 0–50 eV with 0.5 eV steps, and I–V sweep rate ~5 mV/s at room temperature.

**Figure 12:**
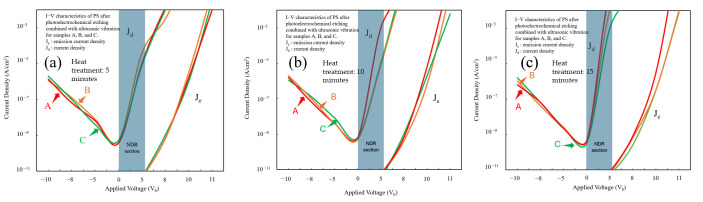
(**a**) I–V characteristics of the NPS device fabricated by 1310 nm photoelectrochemical etching after 5 min thermal treatment; (**b**) I–V characteristics of the NPS device after 10 min thermal treatment, showing a gradual reduction in the NDR region; (**c**) I–V characteristics of the NPS device after 15 min thermal treatment with weakened quantum confinement and emission efficiency.

Three groups of samples were tested three times at different heat treatment durations. The resulting curves depicted in Figure 12a–c illustrate the I–V characteristics of PS devices fabricated using 1310 nm laser-assisted PEEU and liquid-phase bonding. All three curves exhibit typical diode conduction behavior in the low-bias region (–10 V to 0 V). An NDR region appears between 0 V and 5 V, characterized by a decrease in current as voltage increases, indicative of a nonlinear current response. This NDR phenomenon can be attributed to carrier trapping and single-electron effects within the PS nanostructure. When the applied bias reaches a critical value, electron localization within the quantum confined nanopores restricts carrier transport, causing a brief drop in current. As the bias continues to increase, the trap states are saturated, again enhancing carrier tunneling, and the current resumes its upward trend, thus yielding a characteristic I–V reversal curve. As shown in Figure 12a, sample A (subjected to a 5 min heat treatment) exhibits the most pronounced NDR curve, with an initial bias voltage between approximately 4.8 and 5.2 V. As the heat treatment time increases, as shown in Figure 12b,c for samples B and C, respectively, the NDR region gradually evens out and disappears, demonstrating the significant influence of the heat treatment on the PS nanostructure. Such treatment promotes inter-pore recrystallization and partial oxidation of the pore walls, obscuring localized energy levels and diminishing the trap density, thereby attenuating quantum confinement effects. While this structural homogenization improves conductive stability, it concurrently reduces carrier recombination between localized energy levels, resulting in a less pronounced NDR region, as shown in Figure 12c for sample C. At higher bias (>5 V), current density rises rapidly into a J_e_ dominated region. A comparison of curves A through C shows that the overall J_e_ value decreases with increasing heat treatment time, signifying diminished photoelectric conversion efficiency and carrier injection capability due to increased pore size and wall thickness, which weakens the localization of photogenerated carriers and reduces their dispersion.

Emission and carrier injection efficiencies were quantified by defining the emission efficiency as *η* = J_e_/J_d_ [39]. The results show a substantial decrease in η as the duration of heat treatment increases from 5 to 15 min, indicating that high-temperature processing enlarges and compacts the pore structure, consequently reducing carrier confinement and luminescence efficiency.

On the whole, the findings indicate that the quantum confinement effect is relatively weak in PS structures etched using a 1310 nm long-wavelength laser. Longer heat treatment durations reduce the NDR characteristics, emphasizing the roles that pore size and surface state regulation plays in carrier confinement and NDR behavior. These results confirm that the NDR characteristics of NPS devices etched using a long-wavelength laser (1310 nm) are primarily governed by carrier confinement and the structural homogenization effect caused by heat treatment, rather than solely by the voltage–current response.

After PEEU processing, the I–V curves reveal a marginal increase in onset voltage from sample A to sample C, correlating with enhanced surface oxidation and densification resulting from increased heat treatment duration. Sample A exhibits the lowest onset voltage, suggesting easier carrier injection and fewer interfacial defects. Sample B demonstrates a balanced relationship between onset voltage and emission efficiency, exhibiting moderate porosity and optimal heat treatment conditions, resulting in relatively stable luminescence performance. After 15 min of heat treatment, sample C exhibits a slightly higher onset voltage but markedly enhanced PL efficiency at high bias, showcasing excellent carrier capture and recombination characteristics.

These observations point to enhanced carrier confinement in the NPS structure and reduced non-radiative recombination at the interface, ultimately delivering superior overall efficiency, as summarized in Table 2.

## 4. Conclusions

This study employed a combination of laser irradiation, ultrasonic-assisted etching, and APTES-based liquid-phase bonding to significantly enhance etching uniformity, interfacial bonding integrity, and luminescence performance in the NPS structure. Experimental data confirmed the pivotal role of ultrasonic vibration in improving etching stability by removing hydrogen bubbles and accelerating reactant diffusion in the etched area, thereby mitigating local etching inhibition caused by B–Si aggregation. Laser-assisted carrier excitation further augmented the BEA effect. At a 10 V bias, NPS diodes fabricated using the PEEU process combined with APTES-based liquid-phase bonding achieved an average external quantum efficiency of 7.3%, nearly 2.5 times that of samples prepared by conventional photoelectrochemical etching. A 15-min heat treatment following laser-assisted ultrasonic etching yielded an approximately 180% increase in PL intensity, confirming that quantum confinement substantially enhances radiative recombination efficiency. The resulting NPS layer displayed improved PL and stable EL under low bias conditions (0–5 V), accompanied by pronounced NDR, indicating efficient quantum tunneling and carrier recombination within the NPS diode structure. Additionally, APTES-mediated liquid-phase bonding established covalent Si–O–Si bonds between the P-type and N-type PS layers without introducing an intermediate SiO_2_ barrier, significantly improving carrier injection efficiency and minimizing interfacial recombination losses. Collectively, this work presents a novel methodology for achieving tunable wavelength emission and enhanced electronic properties in NPS devices.

## Figures and Tables

**Table 1:** Surface roughness parameters of PS at different etching times derived from AFM image analysis.

Sample	Scan Size	z Range (Approx.)	Estimated Ra (nm)	Estimated Rq (nm)	Estimated Rz (nm)	Estimated Rmax (nm)
5 min	0.5 × 0.5 µm	0.3–6.1 nm	1.0–2.5	1.2–3.2	4.5–6.0	~5.8
10 min	3 × 3 µm	21.3–42.7 nm	6.0–12.0	7.5–15.5	18–30	~21.4
15 min	5 × 5 µm	48–96 nm	12–25	15–32	45–85	~48

**Table 2:** Comparison of I–V characteristics and emission efficiency of NPS emitters under 1310 nm laser-assisted PEEU treatment.

Emitter Sample	PEEU Using Laser	Onset Voltage (V)	Avg η (%) at V_b_ = 10 V
A sample	1310 nm	5.45 ± 0.05	6.9
B sample	1310 nm	5.55 ± 0.05	7.1
C sample	1310 nm	5.65 ± 0.05	7.3

## Data Availability

Data are contained within the article.
